# A systematic study on the binding affinity of SARS-CoV-2 spike protein to antibodies

**DOI:** 10.3934/microbiol.2022038

**Published:** 2022-12-26

**Authors:** Ke An, Xiaohong Zhu, Junfang Yan, Peiyi Xu, Linfeng Hu, Chen Bai

**Affiliations:** 1 Warshel Institute for Computational Biology, School of Life and Health Sciences, School of Medicine, The Chinese University of Hong Kong, Shenzhen, Shenzhen, 518172, Guangdong, People's Republic of China; 2 School of Chemistry and Materials Science, University of Science and Technology of China, Hefei, Anhui, 230026, P. R. China; 3 Chenzhu (MoMeD) Biotechnology Co., Ltd, Hangzhou, Zhejiang, 310005, P.R. China

**Keywords:** SARS-CoV-2, variant, antibody, binding affinity, immune evasion

## Abstract

The COVID-19 pandemic has caused a worldwide health crisis and economic recession. Effective prevention and treatment methods are urgently required to control the pandemic. However, the emergence of novel SARS-CoV-2 variants challenges the effectiveness of currently available vaccines and therapeutic antibodies. In this study, through the assessment of binding free energies, we analyzed the mutational effects on the binding affinity of the coronavirus spike protein to neutralizing antibodies, patient-derived antibodies, and artificially designed antibody mimics. We designed a scoring method to assess the immune evasion ability of viral variants. We also evaluated the differences between several targeting sites on the spike protein of antibodies. The results presented herein might prove helpful in the development of more effective therapies in the future.

## 1. Introduction

The COVID-19 pandemic poses a significant challenge to global public health. In the past two years, SARS-CoV-2 has spread to five continents resulting in more than six million deaths (by 14 September 2022) [1]. Cambridge University estimated that the pandemic would cost around 82 trillion dollars to the global economy over five years [2]. Effective and reliable prevention and treatment methods are urgently demanded to defeat the virus effectively. As of 12 September 2022, more than 12 billion vaccine doses have been administered worldwide [1]. By the end of 2021, eight anti-SARS-CoV-2 drugs based on therapeutic neutralizing antibodies (nAbs) have been approved under Emergency Use Authorization (EUA) by the US Food and Drug Administration (FDA) and/or European Agency of Medicines (EMA) [3]. However, the virus is evolving continuously, giving rise to numerous variants [4]. Some variants (such as Alpha, Beta, Gamma, Delta, and Omicron) with enhanced virulence are defined as variants of concern (VOCs) by the World Health Organization (WHO) [5]. Recently, Omicron has evolved into multiple sub-variants: BA.2, BA.3, BA.4, and BA.5 [5]. The viral variants have, in turn, raised concerns about the effectiveness of the currently available vaccines for coronavirus [6]–[8]. Many studies have reported the high immune evasion ability (IEA) of the Omicron variant and its sub-variants [9]–[15]. Therefore, it is crucial to systematically analyze the effect of mutations in the spike protein on antibody binding in order to guide the design of broad-spectrum antibodies.

The receptor-binding domain (RBD) of the SARS-CoV-2 spike glycoprotein is the target of most current clinical antibodies [3],[16]. The spike protein is a homotrimer located on the surface of the virus membrane that mediates entry of the virus into the host cells via interaction between the receptor binding motif (RBM) of RBD and the human angiotensin converting enzyme 2 (ACE2) receptor [17]. Typically, RBD is buried on the surface of the spike protein. In the presence of ACE2, the conformation of the spike protein shifts to an opening state [18]. This conformational shift brings RBD to the “up” conformational state, and the RBM is exposed on its top surface for ACE2 binding [17]. Most antibodies target the epitopes that overlap with the RBM and interfere with viral infection by blocking the binding of ACE2 to the spike protein [16]. In addition, some antibodies also bind to the side surface of RBD [3],[16]. Mutations in the viral variants weaken the interactions between antibodies and their target epitopes on the variants, thus allowing the immune evasion of the virus. The Omicron variant exhibits low binding affinity to most of the currently available SARS-CoV-2 neutralizing antibodies [9],[12].

Computational biology provides powerful tools to study the binding affinity of the spike protein to various kinds of antibodies based on an energetic perspective. Our previous studies explored the structure/energy basis of spike-ACE2 interactions [19] and the effects of mutations on receptor binding in some variants [20]. We also predicted important mutations, such as N501Y, Q493R, and Q498R, appearing in the subsequent VOCs [19],[20]. These works confirmed the validity of our approach.

In this study, we first constructed the structural models of the spike-antibody complexes based on the experimental structures. Then, a systematic binding free energy (ΔG_binding_) and binding free energy change (ΔΔG_binding_) were assessed to evaluate the differences in the affinity of eight nAbs (AZD1061, AZD8895, CT-P59, LY-COV555, LY-COV016, REGN10933, REGN10987, and S309) to the spike proteins of eight viral variants (Alpha, Beta, Gamma, Delta, Omicron BA.1, Omicron BA.2, Omicron BA.3, and Omicron BA.4; the spike proteins of BA.4 and BA.5 harbored the same mutations). Through the analysis of ΔΔG_binding_ values, we designed a scoring method to assess the IEA of viral variants. Next, we analyzed the differences in ΔG_binding_ and ΔΔG_binding_ during the binding of the ACE2 receptor with two patient-derived antibodies (P22A and 510A5) and an artificially designed antibody mimic (AHB2). We also discussed the possible directions for future antibody design. Our results could help provide valuable insights into developing more effective therapeutic methods for the eradication of SARS-CoV-2 and its variants.

## 2. Materials and methods

### 2.1. Modeling the complex structures

The wild-type spike/ACE2 complex structure was determined by high-resolution cryogenic electron microscopy (cryo-EM) (PDB ID: 7DF4) [18]. Two mutations of the Omicron variant (N679K and P681H) locate in the fragment that is omitted from the experimental structure. Then the missing fragment was repaired using Modeller [21]. In addition, structural data showed that the RBD of the spike protein of the Omicron variant does not exhibit any significant conformational difference from that of the wild-type [22]. Therefore, we used the wild-type structure as the first-order approximation to predict the effects of mutations in the spike protein. The mutations of the eight variants were introduced into wild-type spike protein using PyMOL. Information about the variants of SARS-CoV-2 used in this work is shown in Table 2. After removing the ACE2 receptor from the complex structure, we obtained the isolated spike protein structure. The structures of antibodies and their interactive models were extracted from known structures, which are as follows:

**Table 1.** Information about antibodies.

	Name	PDB ID	Released Date	Epitope regions	Source
Neutralizing antibodies	AZD1061	7L7E	2021-09-01	Top	Neutralized wild-type SARS-CoV-2 virus, the wild type [23],[24]
	AZD8895	7L7D	2021-09-01	Top	Neutralized wild-type SARS-CoV-2 virus, the wild type [23],[24]
	CT-P59	7CM4	2021-01-20	Top	The peripheral blood mononuclear cells of a SARS-CoV-2 convalescent patient, the wild type [25],[26]
	LY-CoV016	7C01	2020-05-27	Top	a convalescent COVID-19 patient from China, the wild type [27]
	LY-CoV555	7KMG	2021-01-27	Top	Isolated from a convalescent COVID-19 patient from North America, unknown type [28]
	REGN10933	6XDG	2020-06-24	Top	Regeneron's VelocImmune® human antibody mouse platform, the wild type [29]
	REGN10987	6XDG	2020-06-24	Top	B cells of human donor previously infected with SARS-CoV-2, the wild type [29]
	S309	7TN0	2022-02-02	Side	Isolated from a subject who recovered from a SARS-CoV infection in 2013 [30],[31]
Patient-derived antibodies	P22A	7CHS	2021-05-19	Top	Isolated from SARS-CoV-2 infected patients, the wild type [32]–[34]
	510A5	7WS7	2022-06-01	Side	COVID-19 convalescent blood samples collected within a 2-month window post discharge, unknown type [35],[36]
Artificially designed antibody mimic	AHB2	7UHB	2022-06-08	Top	

**Table 2.** Information about SARS-CoV-2 variants.

WHO Label	PANGO Lineage	Date first detected	GenBank Accession NO.
Alpha	B.1.1.7	2020-09	OV054768.1
Beta	B.1.351	2020-10	OX003129.1
Gamma	P.1	2020-12	OX000832.1
Delta	B.1.617.2	2020-12	OK091006.1
Omicron	BA.1	2021-11	OX315743.1
Omicron	BA.2	2021-11	OX315675.1
Omicron	BA.4	2022-02	OP093374.1

### 2.2. Calculating the binding free energy change

The all-atom structures were converted into CG representation and subjected to extensive relaxation (11000 steps, 0.0001 ps step-size) under the temperature of 50 K. During relaxation, one structure was obtained every 1000 steps. Finally, we got a conformational trajectory comprising 11 structures. All these structures were used for energy evaluation. Our CG model, which was adapted from previous studies [37]–[39], focused on the precise treatment of the electrostatic charges and was sensitive to the charge distribution of the protein ionized groups. Hence, before energy evaluation, a Monte Carlo proton transfer (MCPT) method [38] was used to determine the charge states of the residues in each structure. During MCPT, protons were “jumped” between ionizable residues, and a standard Metropolis criterion was utilized to calculate the acceptance probability. The total CG folding free energy ($\Delta G_{fold}$) was calculated according to the following formula:

$$\Delta G_{fold}=\Delta G_{main}+\Delta G_{side}+\Delta G_{main-side}=c_{1}\Delta G_{side}^{vdw}+c_{2}\Delta G_{solv}^{CG}+c_{3}\Delta G_{HB}^{CG}+\Delta G_{side}^{elec}+\Delta G_{side}^{polar}+\Delta G_{side}^{hyd}+\Delta G_{main-side}^{elec}+\Delta G_{main-side}^{vdw}$$ (1)

In this formula, the CG folding free energy ($\Delta G_{fold}$) consists of 3 parts: the main chain free energy ($\Delta G_{main}$), the side chain free energy ($\Delta G_{side}$), and the free energy of main-side interactions ($\Delta G_{main-side}$). These three parts can also be divided into 8 terms: side chain van der Waals energy ($\Delta G_{side}^{vdw}$), main chain solvation energy ($\Delta G_{solv}^{CG}$), main chain hydrogen bond energy ($\Delta G_{HB}^{CG}$), side chain electrostatic energy ($\Delta G_{side}^{elec}$), side chain polar energy ($\Delta G_{side}^{polar}$), side chain hydrophobic energy ($\Delta G_{sode}^{hyd}$), main chain/side chain electrostatic energy ($\Delta G_{main-side}^{elec}$), and main chain/side chain van der Waals energy ($\Delta_{main-side}^{vdw}$). The scaling coefficients c_1_, c_2_, and c_3_ were set as 0.10, 0.25, and 0.15, respectively. The energy reached equilibrium after 10000 steps due to the large size of the protein. Therefore, the average energy of the last two structures was used as the final energy. All relative calculations were performed using the Molaris-XG package [40],[41].

The binding energies for spike-antibody complexes ($\Delta G_{binding}$) were calculated using the following formula:

$$\Delta G_{binding}=G_{spike-antibody}-G_{spike}-G_{antibody}$$ (2)

In this formula, the terms on the right represent the CG folding free energy of the spike-antibody complex ($G_{spike-antibody}$), the CG folding free energy of the isolated spike ($G_{spike}$), and the CG folding free energy of isolated antibody ($G_{antibody}$), respectively.

To calculate the binding free energy change ($\Delta \Delta G_{binding}$) of the variant spike-antibody complex, the following formula was used:

$$\Delta \Delta G_{binding}=\Delta G_{binding_{variant}}-\;\Delta G_{binding_{wildtype}}$$ (3)

In this formula, the terms on the right represent the binding free energy of the variant spike to an antibody ($\Delta G_{binding_{variant}}$) and the binding free energy of the wild type spike to the same antibody ($\Delta G_{binding_{wildtype}}$), respectively. The lower the $\Delta \Delta G_{binding}$, the higher the stability.

### 2.3. Calculating the normalized score

The eight variants were considered as the set *V*, and the eight antibodies were considered as the set *A*. For each pair of a variant and an antibody, we calculated ΔΔG_binding_ according to the method described in Section 4.2. All ΔΔG_binding_ values were considered as the set $G=\left\{\Delta \Delta G_{ij}|i\in V,j\in A\right\}$. Each ΔΔG_binding_ value was normalized using the following formula:

$$Normalize\left(\Delta \Delta G_{binding}\right)=\;\frac{\Delta \Delta G_{binding}-Min(G)}{Max\left(G\right)-Min(G)}$$ (4)

In this formula, the terms on the right represent the value would be normalized (ΔΔG_binding_), the minimum value of all ΔΔG_binding_ values (Min(G)), and the maximum value of all ΔΔG_binding_ values (Max(G)).

The IEA score of each variant (*S_i_*) was calculated using the following formula:

$$S_{i}=\frac{\sum_{j}^{j\in A}Normalize(\Delta \Delta G_{binding})}{8},i\in V$$ (5)

In this formula, the terms on the right represent each variant belong to the set *V* (i) and each antibody belong to the set *A* (j). The lower the *S_i_*, the weaker the immune escape ability.

### 2.4. Analyzing the interaction surface

The interfaces between antibodies and the spike protein were analyzed using PDBePISA [42].

## 3. Results

### 3.1. Assessing the immune evasion ability of variants

**Figure 1.**
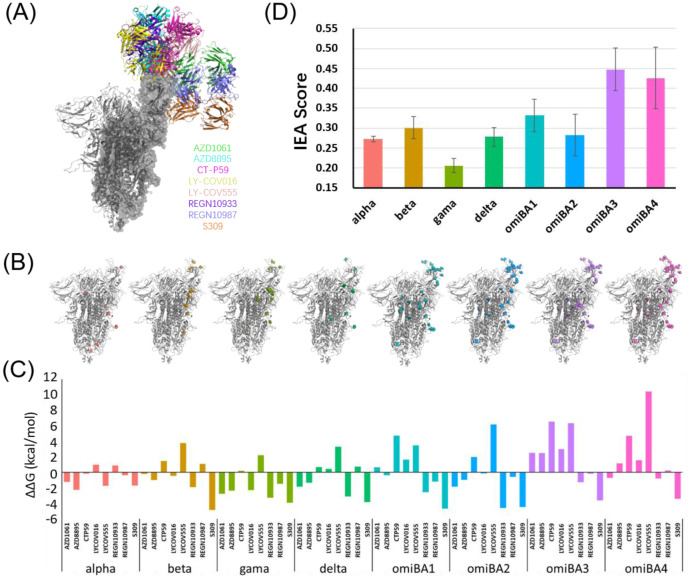
(A) The eight nAbs bind to the spike protein with different poses; (B) Mutations in the SARS-CoV-2 spike of each variant, and the mutated sites are highlighted by colors; (C) The binding free energy change (ΔΔG_binding_) of each combination of variant and antibody; (D) The normalized score of the IEA of variants, the error bar represents the variance in the values of a variant.

The eight nAbs selected for this study recognize different antigenic sites in the RBD of the spike protein of SARS-CoV-2 (Figure 1A). These antibodies can be divided into two groups based on whether or not they can block the spike protein's binding to ACE2 [43]. The first groups comprised seven nAbs (AZD1061, AZD8895, CT-P59, LY-COV555, LY-COV016, REGN10933, and REGN10987) binding to the epitopes that overlap with the RBM of the top site of RBD (Figure 1A), which is also the primary binding site of the ACE2 receptor. The virus cannot be recognized and activated in the presence of these antibodies, thus losing the ability to infect human cells [16]. The second group consisted of the S309 antibody that binds to the side sites of the RBD (Figure 1A). S309 targets non-RBM sites and hence does not block the binding of the spike protein to the ACE2 receptor. Therefore, the S309 binding is not dependent on the conformation of the spike protein and is hypothesized to access all three epitopes of the spike trimer and sterically shield the engagement of ACE2 to block the viral infection [16].

The mutations in SARS-CoV-2 variants result in changes in the binding affinities of their spike proteins to the eight nAbs. The eight chosen variants' spike proteins have mutations that were primarily clustered in the RBM region (Figure 1B), which might enhance the binding affinity of their spike proteins to the ACE2 receptor [20],[44]. Meanwhile, such mutations have been shown to weaken the spike-antibody interaction and interfere with antibody recognition, thus conferring IEA to viral variants [9]–[11],[43]–[47]. Therefore, the ΔΔG_binding_ induced by the mutations on the spike protein can be used to determine the IEA of variants.

We calculated the binding free energies of the wild-type (ΔG_binding-wt_) spike and each variant spike protein (ΔG_binding-variant_) with each therapeutic nAb. Then, we calculated the binding free energy change (ΔΔG_binding_ = ΔG_binding-variant_ - ΔG_binding-wt_, see Methods). In total, the more recent the variants of the spike protein are, the lower binding ability they have to nAbs (Figure 1C). In at least one variant, a reduction in the binding affinity of the nAbs belonging to the first group to the RBD was observed (Figure 1C). For instance, we noticed a mild decrease in the binding affinity of RGEN10933 to the alpha variant, but increases in the other seven variants (Figure 1C). However, the binding affinities of the spike proteins of all eight variants to S309 were enhanced compared to the wild-type spike. (Figure 1C, Table S1). This finding might be attributed to the fact that the binding epitopes of S309 are evolutionarily conserved across several sarbecoviruses, including SARS-CoV [43],[47]. Our findings that most nAbs show weaker binding affinity to the Omicron variants except for S309 were consistent with recent studies [10],[11]. To intuitively measure the IEA of viral variants, we designed a scoring system based on the ΔΔG_binding_ calculations. The ΔΔG_binding_ value of each variant-antibody combination was normalized into the range [10],[11] by the min-max feature scaling method. The IEA score assigned to a variant was the average of its eight normalized values (corresponding to eight antibodies). Our findings showed that the recently evolved sub-lineages of Omicron, BA.3 and BA.4, possess the most robust ability to evade antibody-induced neutralization (Figure 1D, Table S2). According to the variance of all ΔΔG_binding_ values of a variant (the error bar in Figure 1D), we find the IEA score is sensitive to a single value. For instance, the variance of BA.3 is lower than that of BA.4. Combined with the ΔΔG_binding_ calculations, we find that the BA.3 weakens the affinity with most of the nAbs, while the BA.4 exhibits the most prominent reduction in binding affinity to LY-COV555 in all antibodies, which might be contributed to that fact that BA.4 has an additional L452R mutation located on the interface between LY-COV555 and spike (Figure 1C, Figure S1). The substitution of Leucine (uncharged and small side chain) with Arginine (positive charged and large side chain) may disrupt the electrostatic interaction or result in steric hindrance to affect the binding between LY-COV555 and the spike (Figure S1). The IEA scores of these variants indicated that the IEA of SARS-CoV-2 is reinforced in the newly emerging variants (Figure 1D) , which is consistent with the trend researchers observed [48]. Thus, the IEA scoring system can also be used to assess the immune escape capability of new viral variants that might evolve in the future.

### 3.2. Analyzing the binding affinity of the spike proteins to P22A and 510A5

**Figure 2.**
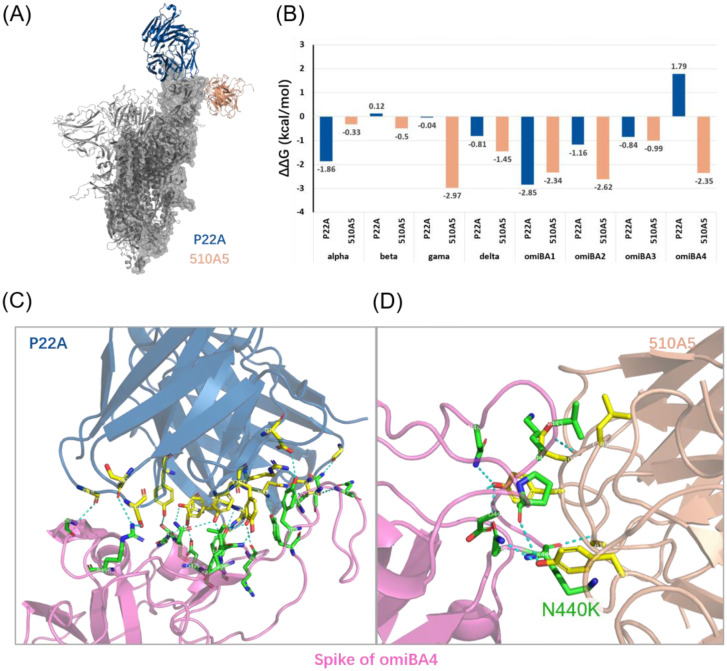
(A) The two patient-derived antibodies (P22A and 510A5) bind to different sites on the spike protein; (B) The binding free energy change (ΔΔG_binding_) for each combination of variant and antibody; (C, D) The residues contribute to the formation of hydrogen bonds when antibodies bind to spike; spike residues are shown in green, antibody residues are shown in yellow, and the hydrogen bonds are shown in cyan.

To further investigate the differences between the top and side epitopes of the spike RBD, we analyzed the binding energies of the spike proteins complexed with two patient-derived antibodies, P22A and 510A5. The P22A antibody was isolated from a patient infected with the early Wuhan strain (wild-type) [49],[50]. P22A can potently neutralize the wild-type spike [49],[50], but so far, no data has shown its ability to bind to the spike protein of variants. P22A binds to the top side of RBD (Figure 2A) and causes massive spatial clashes with ACE2 (approximately 2000 Å^3^) [49],[50]. The 510A5 antibody was isolated from a patient infected with an unknown type of SARS-CoV-2 (not reported in the original paper), but exhibiting binding affinity to the wild-type, Delta, and Omicron variants [51]. It can bind to both the top and side sites of the RBD of the wild-type spike protein but can only bind to the side sites in the Omicron variant (Figure 2A), resulting in clashes with ACE2 [51].

We calculated the ΔG_binding_ and the ΔΔG_binding_ values for WT and all variants. Despite the P22A antibody being produced by induction of wild-type virus particles, it exhibits lower ΔG_binding_ than the 510A5 for all eight variants (Figure S2). This finding might be ascribable to the difference between the top and side binding sites of RBD. Therefore, we performed the interface analysis of the antibody-spike complex structures with respect to these two antibodies. Our results showed that P22A has a larger interface area (1102.1 Å^2^ vs. 664.7 Å^2^), interacts with more spike residues (41 vs. 18), and forms more hydrogen bonds (22 vs. 7) than 510A5 (Figure 2C, D, Table S3). The ΔΔG_binding_ values showed that the binding affinity of 510A5 to the spike protein is enhanced for all variants. This result was comparable to that observed for S309 (Figure 1C). Many studies have reported the high immune evasion ability of the Omicron variant and its sub-variants. Therefore, we expected that the omicron BA.1, BA.2, BA.3, and BA.4 should have poor binding affinity to P22A compared to the wild type. However, our simulation results show that the omicron BA.1, BA.2, and BA.3 exhibit increased binding affinity to P22A, except for the omicron BA.4 (1.79 kcal/mol, Figure 2B) which presents a prominent decrease in binding affinity. These results may indicate that, for antibody P22A, not all the Omicron sub-variants exhibit high immune escape ability to antibody P22A, but the BA.4 is the most transmissible virus variant. The interface analysis showed that, compared to the wild-type, the spike-510A5 interface area increases while the spike-P22A interface area decreases for the Omicron BA.4 variant (Table S3). In the case of 510A5, the increase in interface area can be credited to the N440K mutation located on the side site (area increases from 137.9 to 148.6 Å^2^) and the residue N439 that is near N440K (area increases from 8.9 to 13.8 Å^2^). These findings indicated that the epitopes on the side sites of RBD are more resistant to the enhanced IEA of viral variants.

### 3.3. Analyzing the binding affinity of the spike protein to AHB2 and ACE2

**Figure 3.**
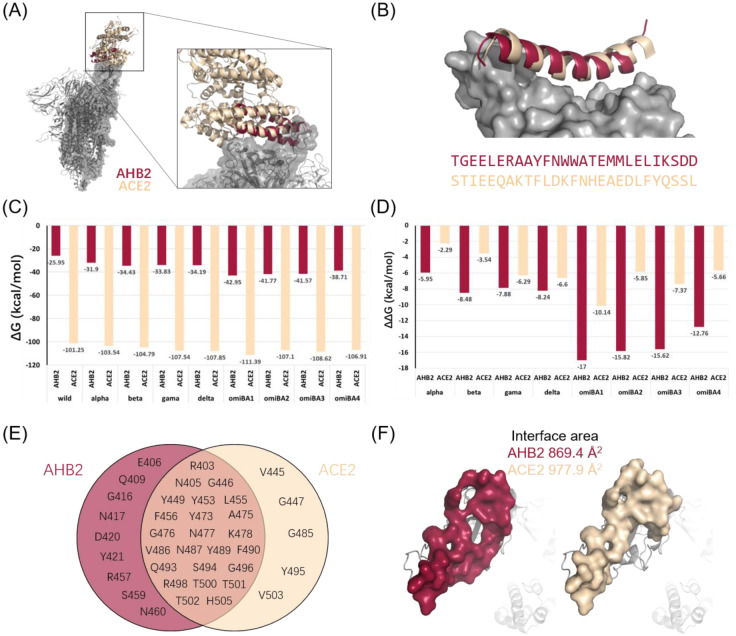
(A) The structures and interaction modes of AHB2 and ACE2; (B) The structure and sequence alignments of the main RBD-interacting helix of the two proteins; (C) The binding free energy (ΔG_binding_) of each combination of variants and the two proteins; (D) The binding free energy change (ΔΔG_binding_) of each combination of variants and the two proteins; (E, F) The key residues and interaction surface of the spike protein contributed to the binding of AHB2 and ACE2.

Except for nAbs, engineered ACE2 traps, such as protein mimics, are also promising therapeutic tools to counter SARS-CoV-2 [52],[53]. Baker et al. designed a lot of minibinder proteins as ACE2 traps to block the interaction between the spike protein and ACE2 [54],[55]. To assess the effectiveness of minibinders in counteracting immune escape of viral variants, it is best to compare the binding affinities of them with that of ACE2. We performed structural and energy analysis of AHB2, which is representative of these minibinder proteins. The binding mode and the helical structure of AHB2 that interacts with the RBD domain of the spike protein are similar to those of ACE2 (Figure 3A, B). However, despite being an ACE2 mimic, the main RBD-interacting helix of AHB2 has low sequence similarity with ACE2 (Figure 3B). We then calculated the ΔG_binding_ of the AHB2 or ACE2-spike protein complex. Our results showed that the binding affinities of wild-type and all variants to ACE2 were much stronger than those to AHB2 (Figure 3C). The finding might be attributed to the fact that ACE2 is a natural receptor that is more prone to bind to spike. The area of the ACE2-spike interface was larger than that of the AHB2-spike interface (Figure 3F). We also found a substantial enhancement in the binding affinity of ACE2 to the spike protein of more recently evolved variants (Figure 3C, D), which might explain the high transmissibility of the Delta and Omicron variants [20],[22],[56]–[58]. However, the variants exhibited a higher binding affinity to AHB2 (Figure 3C, D), implying that AHB2 could resist the neutralization potency reduction induced by mutations in the spike protein. This finding was consistent with previous experimental results showing that AHB2 can effectively neutralize the Delta and Omicron BA.1 variants [55]. In addition, our results also showed that AHB2 exhibited improved affinity to other sub-lineages of the Omicron variants, that is, BA.2, BA.3, and BA.4 (Figure 3D). We identified the residues of the spike protein of Omicron BA.4 that participates in the interaction with AHB2 and ACE2. Most residues that involved in the complex formation were identical, proving that AHB2 binds to the same sites as ACE2 (Figure 3E). Although the area of the ACE2-spike interface is larger, five additional spike residues interact with AHB2 (Figure 3E, Table S4). We speculated that AHB2 resisted the neutralization potency reduction of variants by interacting with as many spike residues as possible. In summary, these results highlighted the potential of ACE2 mimics as antiviral therapeutic tools to mitigate the rapidly evolving pandemic.

## 4. Discussion and conclusions

Using structural modeling and computational biology methods, we systematically analyzed the binding affinity of the spike proteins of eight SARS-CoV-2 variants to three groups of antibodies: monoclonal nAbs for therapeutic, antibodies isolated from patients who were infected with the virus, and artificially designed antibody mimics. The immune evasion of newly emerging viral variants is a significant issue at present, with concrete manifestation in the reduced binding affinity of mutant spike proteins to antibodies. We designed a scoring system based on the ΔΔG_binding_ values to evaluate the IEA of variants. This method can also be applied to evaluate an antibody's broad-spectrum effectiveness, thus playing an important role in designing antibodies targeting the spike protein.

The antibodies selected for this study bound to either the top or the side sites of the RBD of spike protein, neutralizing the coronavirus in multiple ways [16]. As the top sites overlap with the RBM, the antibodies bind to this site directly block the interactions between the spike protein and the ACE2 receptor. Our results showed that the antibodies bound to the top sites exhibited a higher binding affinity than those that bound to the side sites. This might be attributed to the larger interaction interface and the formation of more hydrogen bonds during the interactions with the top sites. However, mutations also tend to aggregate at the top sites. Subsequently, the antibodies that bind to the top sites are more susceptible to variant evasion than those that bind to the side sites. Since the side sites are more evolutionarily conserved, the antibodies that target these sites show comparable binding affinity to the spike proteins across different variants.

Our results reflect a comprehensive conclusion that the Omicron sub-variants have higher immune escape ability than other variants of SARS-CoV-2. Actually, the binding affinity and the interaction mode between the spike protein and antibodies are more complicated than we discussed in this work. As we discussed in section 3.2, to the specific antibody P22A, the early Omicron sub-variants (BA.1 and BA.2) exhibit stronger binding affinities than the alpha, beta, gamma, and delta variants. These antibodies may have some important interaction modes that could help us develop new therapeutic antibodies. In this study, we only calculated the binding energies and interaction modes of two specific antibodies, P22A and 510A5. More representative antibodies should also be included in our studies in the future, which may give rise to a new avenue for designing effective and stable antibodies.

Steric effects triggered by antibodies play an important role in blocking the binding of ACE2 to the spike protein. Previous studies have shown that the 510A5 antibody cannot effectively neutralize the Omicron spike protein, even though it binds to the side sites of its RBD [51]. When binding to the side sites, this antibody cannot generate sufficient steric hindrance to block the interaction between ACE2 and the spike protein [51]. These findings suggest that side-site targeting antibodies might fail to block ACE2-spike interaction, although the viral variant does not readily evade their binding. Considering the advantages and drawbacks of the top and side sites, exploiting the combined advantages conferred by the antibodies that bind to these two types of sites could lead to the development of better therapeutic approaches.

In addition to the top and side sites of RBD, antibodies targeting the fusion peptides of the spike protein have also been reported in recent studies [59],[60]. After binding to the ACE2 receptor, the spike protein is divided into two parts, S1 and S2, by the membrane enzyme transmembrane serine protease 2 (TMPRSS2) or endosomal cathepsins [61],[62]. Then, the S1 subunit is shed, and the fusion peptide is exposed, leading to the insertion of the fusion peptide into the cell membrane, which results in viral fusion [61],[62]. Some motifs of the fusion peptide are highly conserved. Thus, the antibodies targeting these motifs can neutralize several viral variants [59],[60]. Therefore, fusion peptides can also be used as an essential epitope in developing effective antibodies. In future studies, we will analyze the binding affinities of the existing antibodies that target the fusion peptide.

Overall, our study presented a systematic analysis of the binding affinity of various antibodies to the spike proteins of various variants. We compared the effects of two types of RBD binding sites on antibody binding and neutralization evasion of viral variants. Our findings can be used to design more effective broad-spectrum antibodies or mimics against viral agents.
