# Supplementary material for: A systematic study on the binding affinity of SARS-CoV-2 spike protein to antibodies
Source: AIMS Microbiol. 2022 Dec 26;8(4):595–611. doi: 10.3934/microbiol.2022038 (PMC9834082; doi:10.3934/microbiol.2022038)
Supplement: Supplementary file 1 [file microbiol-08-04-038-s001.pdf]

AIMS Microbiology

AIMS Microbiology, 8(4): 595–611.
DOI: 10.3934/microbiol.2022038
Received: 29 September 2022
Revised: 18 December 2022
Accepted: 21 December 2022
Published: 26 December 2022

http://www.aimspress.com/journal/microbiology

Research article

A systematic study on the binding affinity of SARS-CoV-2 spike protein to antibodies

Ke An[1,2], Xiaohong Zhu[1,2], Junfang Yan[1], Peiyi Xu[1], Linfeng Hu[1] and Chen Bai[1,3,*]

[1] Warshel Institute for Computational Biology, School of Life and Health Sciences, School of Medicine, The Chinese University of Hong Kong, Shenzhen, Shenzhen, 518172, Guangdong, People's Republic of China
[2] School of Chemistry and Materials Science, University of Science and Technology of China, Hefei, Anhui, 230026, P. R. China
[3] Chenzhu (MoMeD) Biotechnology Co., Ltd, Hangzhou, Zhejiang, 310005, P.R. China

* **Correspondence:** Email: baichen@cuhk.edu.cn

Supplementary

Table S1. The binding free energy change (ΔΔG, in kcal/mol) of each pair of variant and neutralizing antibody.

	AZD1061	AZD8895	CTP59	LYCOV016	LYCOV555	REGN10933	REGN10987	S309
alpha	-1.24	-2.28	-0.20	0.97	-1.77	0.86	-0.41	-1.72
beta	-0.22	-1.02	1.42	-0.46	3.77	-1.92	1.06	-4.88
gama	-2.78	-2.39	0.17	-2.31	2.19	-3.31	-1.48	-3.95
delta	-1.88	-1.36	0.66	0.42	3.29	-3.13	0.71	-3.84
omiBA1	0.63	-0.39	4.70	1.62	3.46	-2.60	-1.23	-4.70
omiBA2	-1.86	-0.98	1.96	-0.16	6.14	-4.62	-0.60	-4.49
omiBA3	2.48	2.45	6.51	2.97	6.30	-1.29	-0.19	-3.64
omiBA4	-0.74	1.14	4.67	1.55	10.40	-0.80	0.21	-3.45

Table S2. The normalized score of the immune escape ability of variants.

	AZD1061	AZD8895	CTP59	LYCOV016	LYCOV555	REGN10933	REGN10987	S309
alpha	0.24	0.17	0.31	0.38	0.20	0.38	0.29	0.21
beta	0.30	0.25	0.41	0.29	0.57	0.19	0.39	0.00
gama	0.14	0.16	0.33	0.17	0.46	0.10	0.22	0.06
delta	0.20	0.23	0.36	0.35	0.53	0.11	0.37	0.07
omiBA1	0.36	0.29	0.63	0.43	0.55	0.15	0.24	0.01
omiBA2	0.20	0.26	0.45	0.31	0.72	0.02	0.28	0.03
omiBA3	0.48	0.48	0.75	0.51	0.73	0.23	0.31	0.08
omiBA4	0.27	0.39	0.63	0.42	1.00	0.27	0.33	0.09

Table S3. The interface summary of the two antibodies (P22A and 510A5) with the wild-type and omicron BA.4 spike

		P22A		510A5	
		Wild-type	OmiBA.4	Wild-type	OmiBA.4
Heavy Chain	Interface Area (Å^2)	733.2	721.5	452.9	466.2
	Number of H-bonds	16	19	5	5
	Number of spike residues	24	24	12	12
	Number of antibody residues	20	20	13	13
Light Chain	Interface Area (Å^2)	383.4	380.6	182.3	198.5
	Number of H-bonds	4	3	2	2
	Number of spike residues	18	17	6	6
	Number of antibody residues	12	13	6	8

Table S4. The interface summary of the two proteins (AHB2 and ACE2) with the omicron BA.4 spike

	AHB2	ACE2
Interface Area (Å^2)	869.4	977.9
Number of H-bonds	3	8
Number of spike residues	33	29
Number of protein residues	23	29

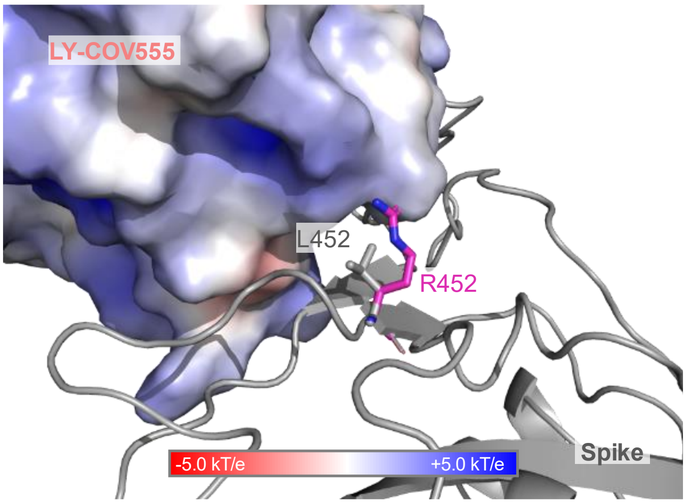

Figure S1. The L452R mutation located on the interface of LY-COV555 and spike. The electrostatic potential surface calculated by APBS of the LY-COV555 antibody is shown.

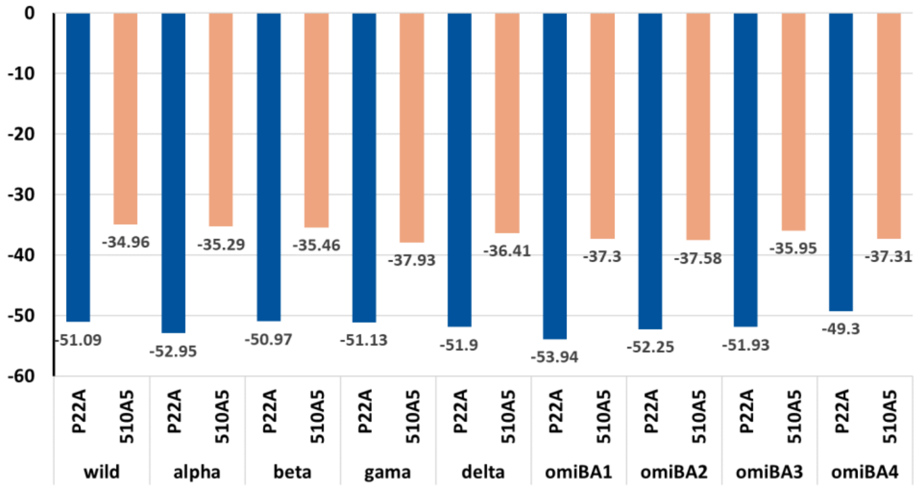

Figure S2. The binding free energy ($\Delta G_{binding}$, in kcal/mol) of the two antibodies (P22A and 510A5) with the spike protein of eight variants.

AIMS Press

© 2022 the Author(s), licensee AIMS Press. This is an open access article distributed under the terms of the Creative Commons Attribution License (http://creativecommons.org/licenses/by/4.0)
